# Self-Learning Microfluidic Platform for Single-Cell Imaging and Classification in Flow

**DOI:** 10.3390/mi10050311

**Published:** 2019-05-09

**Authors:** Iordania Constantinou, Michael Jendrusch, Théo Aspert, Frederik Görlitz, André Schulze, Gilles Charvin, Michael Knop

**Affiliations:** 1 Zentrum für Molekulare Biologie der Universität Heidelberg (ZMBH), DKFZ-ZMBH Alliance, Universität Heidelberg, 69120 Heidelberg, Germany; jendrusch@stud.uni-heidelberg.de (M.J.); f.goerlitz@gmx.net (F.G.); a.schulze@zmbh.uni-heidelberg.de (A.S.); 2 Institute of Microtechnology, Technische Universität Braunschweig, 38124 Braunschweig, Germany; 3 Center of Pharmaceutical Engineering (PVZ), Technische Universität Braunschweig, 38106 Braunschweig, Germany; 4 Developmental Biology and Stem Cells Department, Institut de Génétique et de Biologie Moléculaire et Cellulaire, 67400 Illkirch-Graffenstaden, France; aspertt@igbmc.fr (T.A.); charvin@igbmc.fr (G.C.); 5 Centre National de la Recherche Scientifique, 67400 Illkirch-Graffenstaden, France; 6 Institut National de la Santé et de la Recherche Médicale, 67400 Illkirch-Graffenstaden, France; 7 Université de Strasbourg, 67400 Illkirch, France; 8 Cell Morphogenesis and Signal Transduction, German Cancer Research Center (DKFZ), DKFZ-ZMBH Alliance, 69120 Heidelberg, Germany

**Keywords:** microfluidics, 3D flow focusing, 3D particle focusing, particle/cell imaging, bioMEMS, unsupervised learning, neural networks, variational inference

## Abstract

Single-cell analysis commonly requires the confinement of cell suspensions in an analysis chamber or the precise positioning of single cells in small channels. Hydrodynamic flow focusing has been broadly utilized to achieve stream confinement in microchannels for such applications. As imaging flow cytometry gains popularity, the need for imaging-compatible microfluidic devices that allow for precise confinement of single cells in small volumes becomes increasingly important. At the same time, high-throughput single-cell imaging of cell populations produces vast amounts of complex data, which gives rise to the need for versatile algorithms for image analysis. In this work, we present a microfluidics-based platform for single-cell imaging in-flow and subsequent image analysis using variational autoencoders for unsupervised characterization of cellular mixtures. We use simple and robust Y-shaped microfluidic devices and demonstrate precise 3D particle confinement towards the microscope slide for high-resolution imaging. To demonstrate applicability, we use these devices to confine heterogeneous mixtures of yeast species, brightfield-image them in-flow and demonstrate fully unsupervised, as well as few-shot classification of single-cell images with 88% accuracy.

## 1. Introduction

Phenotypic profiling of cell populations is routinely performed in research and diagnostic laboratories using flow cytometry [1,2,3]. Flow cytometry provides cellular analysis at an unparalleled throughput and allows for the screening of diverse samples and the isolation of cell subpopulations for further study. Standard applications of flow cytometry employ multi-channel fluorescence detection and sample characterization based on light scattering and fluorescence signal intensity, which provide limited spatial resolution [4]. Imaging flow cytometry combines the speed and sample size of flow cytometry with spatial resolution and allows for the acquisition of images and their use for sample characterization and sorting decisions [5]. While imaging flow cytometry gives researchers the opportunity to conduct multiparametric analysis of cell populations based on single-cell images, acquiring high-resolution images at throughputs common in flow cytometry remains challenging. This is primarily due to the difficulty of precisely positioning cells, and the challenges associated with imaging fast moving objects [6].

Recently, there has been a push towards the development of microfluidic-based flow cytometers with the aim to reduce complexity and sample volume and increase accessibility and portability [7,8]. One challenging aspect in the miniaturization of flow cytometers has been the focusing of fast-moving cells in a small, defined volume. Successful 3D hydrodynamic focusing techniques have been demonstrated over the past decade, but many rely on multi-layer structures, incompatible with polydimethylsiloxane (PDMS)-based soft-lithography [9,10,11]. Additionally, particles in these devices are focused to the center of tall microfluidic channels, tens, or hundreds of micrometers away from the microscope slide which is suboptimal for imaging flow cytometry [12]. For better signal-to-noise ratio, it is preferable that particles are positioned as close to the microscope slide as possible, minimizing background fluorescence. More importantly, when the specimen is located far from the surface of the cover slip the resolution obtained using high numerical aperture (NA) oil objectives is impaired. The use of such objectives is required for sensitive fluorescence detection, but resolution decreases with distance due to optical aberrations arising from a difference in refractive index between the immersion liquid (oil) and the sample (particles in aqueous solution) [13,14]. Moreover, due to the limited depth of field in conventional microscopy, particles in imaging microfluidic cytometers need to be focused in a small volume along the z-axis for high resolution imaging [15].

Microfluidic devices designed for imaging flow cytometry have been demonstrated with partial success. Devices that utilize inertial forces (i.e., inertial lift and Dean forces that arise from fluid and particle inertia) for cell positioning have been a popular alternative to sheath flow-based particle steering. The most prominent examples include curved and spiral microchannels, but successful positioning in such devices demands high flow rates that often compromise image quality [16,17]. While imaging technologies for flow cytometry have been suggested, imaging particles moving at high velocities while processing captured images in parallel still remains challenging [6]. Another popular device design used for imaging applications utilizes channel heights that are comparable in size to the particles being imaged. Such forced confinement works well for bigger cells, like blood cells, but it’s prone to clogging, which makes it unsuitable for smaller cells that often tend to aggregate, such as yeast cells [18]. Recently, the first presentation of a microfluidics-based, imaging flow cytometer capable of cell sorting was demonstrated, and it is expected to lead the way for a new field to emerge in microfabrication [19]. Despite the successful demonstration, this system is complicated to set up and operation still requires trained technicians.

Another big challenge associated with high-throughput imaging flow cytometry is the vast amount of complex data collected. As manual analysis of such data sets would be prohibitively slow and laborious, automated image analysis using advanced algorithms is necessary. In the case of standard flow cytometry, event data is low-dimensional and can be analyzed semiautomatically using gating on fluorescence channel intensities and standard non-parametric clustering of gated events [20,21,22]. Here dimensions correspond to meaningful visual features such as cell shape and cell focal plane. These approaches break down in the face of high-dimensional data due to what is known as the ‘curse of dimensionality’ [23]. For complex, high-dimensional data such as single-cell images, distance measures become less useful for clustering and values in single dimensions (i.e., pixel values) become less informative for gating purposes [24]. Using hand-crafted sets of image features, more informative low-dimensional representations of images can be extracted [25,26]. Such features could include texture and image moments, or in more specific cases, cell features like elongation and size. However, these representations are task specific, and may not reflect any kind of biologically relevant properties. To counteract these restrictions, neural networks are becoming popular for the task of learning biologically interpretable classifiers for imaging data based on which different types of cells can be classified [27,28,29]. However, in order to apply neural networks to a classification problem, the data need to satisfy two main conditions: classes of data need to be known, and data acquisition and annotation should be efficient. For example, neural networks can be trained and applied for the classification of single-cell imaging data with known categorical factors of variation (e.g., species, or protein localization) and easily acquired and annotated training data sets. Successful applications of neural network classifiers include imaging flow cytometry, image activated cell sorting [19], and offline analysis of single-cell imaging data [27,29]. Other classifiers such us support vector machines (SVM) have also been similarly used for the offline analysis of single-cell imaging data [30,31]. However, such approaches become difficult to use when it comes to analyzing complex populations of *a priori* unknown factors of variation, or performing classification tasks using limited training data.

The characterization of a data set without the use of training examples is known as unsupervised learning. As fully unsupervised classification is a hard problem, a variety of methods focus on simplifying this task by learning meaningful low-dimensional representations of high-dimensional data [32]. For that reason, neural networks are not trained directly for classification, but on related tasks, where it is possible to generate training data artificially [33,34,35]. A more natural approach to imaging data classification is learning to generate realistic image samples from a data set [36,37,38]. For example, networks can be trained to predict the relationship between rotations, zooms and crops of a given image, or learn to construct realistic images from a low-dimensional representation. This way, the networks learn low-dimensional features relevant to their training data and by extension to downstream classification tasks, without explicitly being trained on annotated examples. Recent approaches further demand low-dimensional representations to be human-interpretable, such that every dimension corresponds to a single factor of variation of the training dataset. For example, training on single cell images should result in a representation, where one dimension corresponds to cell type, another to cell size and yet another to the position of the cell within the image. Such representations are referred to as disentangled representations. Disentangled representations have been shown to be beneficial for classification using very few training examples (few-shot classification) [39]. A subset of unsupervised learning methods known as variational autoencoder (VAE) provide a foundation for learning disentangled representations that are simple to train and implement [40,41,42,43,44,45]. In particular, FactorVAE and related methods explicitly modify the VAE training process to promote more interpretable representations.

In this report, we attempt to bridge the gap between technology and biology and present a self-learning microfluidic platform for single-cell imaging and classification in flow. To achieve 3D flow and particle focusing, we use a simple microfluidic device, based on a variation of the commonly used three-inlet, Y-shaped microchannel. We utilize a difference in the height between sheath and sample inlet to confine heterogeneous cells in a small controllable volume directly adjacent to the microscope cover slide, which is ideal for high-resolution imaging of cells in flow. Even though the device design is conceptually similar to previous designs [46,47,48], controlled 3D hydrodynamic flow focusing has never been fully demonstrated in such devices, nor has particle positioning in focused flow streams been investigated. In this study, we fully characterize different device variations using simulations, and experimentally confirm 3D flow focusing using dye solutions. Additionally, we use a novel, neural network-based regression method to directly measure the distribution of microspheres and highly heterogeneous cells within the focused stream. We confine and image mixtures of different yeast species in flow using bright-field illumination and classify them by species by performing fully unsupervised, as well as few-shot cell classification. To our knowledge, this is the first application of unsupervised learning to classification in imaging flow cytometry.

## 2. Materials and Methods

### 2.1. Device Design and Fabrication

**Figure 1:**
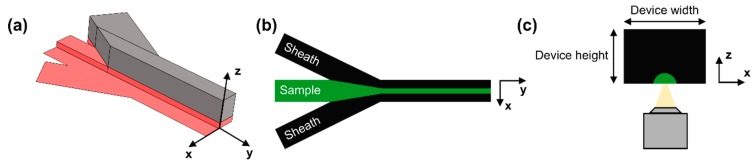
(**a**) Lengthwise 3D device cross-section showing the difference in height between the sheath and sample inlets. Red color is used to show the bottom layer of photoresist, and also the device footprint. The top layer of photoresist is shown in grey (mirror symmetry across the y-axis applies). The difference in height between the sheath inlet and the sample inlet shown is not drawn to scale and only serves as an example; (**b**) Device top view showing the flow focusing mechanism, where the black area is occupied by sheath fluid and the green area is occupied by the sample; (**c**) 2D lengthwise cross-section of the channel (front view) showing sample confinement from both the top and the sides.

To achieve sample flow focusing close to the surface of the microscope cover slip we redesigned a simple microfluidic device based on a variation of the commonly used Y-shaped microchannel (Figure 1) [9,46,47,48]. For the fabrication of the silicon wafer master, we used standard two-layer, SU-8 (MicroChem, Westborough, MA, USA) photolithography [49]. Figure S1a,b show the two layers of photoresist used in the fabrication process. By sequentially combining these two layers, we created a device with three inlets and one outlet, as shown in Figure S1c. The two outer inlets introduce sheath fluids in the device and are taller than the middle inlet, which delivers the sample containing the particles under investigation (i.e., microspheres, cells). The height ratio between the sheath inlets and sample inlet can be controlled by adjusting the height of photoresist and several ratios can be fabricated for testing using the same set of photomasks. Devices for testing were fabricated using single-layer, PDMS-based soft lithography (SYLGARD™ 184 Silicone Elastomer, Dow Chemical, Midland, MI, USA) [50]. Since the difference in inlet height is created at the wafer-level, PDMS devices are fabricated in a single step, with no need of assembling a multi-layer structure.

### 2.2. Flow Focusing Principle

A schematic of the full device geometry is shown in Figure 1a. For better visualization of the difference in height between the sheath inlets and the sample inlet, a lengthwise 3D cross section of the device is provided. Any desired height ratio is possible as long as the layer thickness lies within the resolution of the photoresist and the height:width channel aspect ratio remains lower than 1:10 to ensure that channels will not collapse. The flow focusing mechanism behind these devices is shown in Figure 1b (top view; xy-plane) and Figure 1c (cross section; xz-plane). We use a three-inlet, Y-shaped microchannel to introduce the sample along with two sheath flows. The sample enters the device from the middle inlet (shown in green) and is enfolded by two sheath streams (shown in black, Figure 1b). Due to the occurrence of laminar flow in microchannels, the three streams flow parallel to each other without convective mixing [51]. While 2D sample confinement on the xy-plane has been demonstrated in similar devices numerous times [52,53,54], simultaneous flow confinement along the z-axis (3D flow focusing) has only been briefly investigated [46,47,48]. One example is a paper by Scott et al., where 2D and 3D flow confinement were achieved in devices of similar geometry, but confinement below the sample inlet height required a geometry modification, a step in the outlet channel after the junction [46]. One other example is a paper by Chung et al. where 3D flow confinement was demonstrated successfully, but control over the degree of confinement also required geometry modifications [48]. Flow focusing along the z-axis is illustrated in Figure 1c. As drawn in the figure, due to the difference in height between the sheath and sample inlet, sheath fluids surround sample flow from all sides (black color), constraining it in a small volume close to the microscope cover slide. Here, the final volume occupied by the sample stream merely depends on the sheath-to-sample flow rate ratio, with higher sheath flow rate resulting in further sample confinement in both the xy- and xz-planes for any given device height.

### 2.3. Device Simulations

The device geometry was parameterized within COMSOL Multiphysics^®^, leaving the channel widths, the sample channel height and the sheath fluid channel height as variables. Two-fold symmetry was exploited by simulating only one half of the device split along the x-axis, applying symmetric boundary conditions where appropriate. Steady-state fluid flow through the device was simulated using the computational fluid dynamics (CFD) module, coupled to the transport of diluted species module for all simulations involving fluorescein. We used the particle tracing module for all simulations involving microparticles. Simulations were conducted under the assumption of laminar flow, with no-slip boundary conditions on all walls. Inlets were subjected to laminar inflow constraints, parameterized by the sample flow rate and the sheath flow rate respectively. The outlet pressure was constrained to zero. Fluid parameters were assigned for liquid water at 293 K. For simulations involving fluorescein, the sample inlet was subjected to a concentration constraint, fixing the concentration of fluorescein at the inlet to its experimental value of 1 mM/mL. All other inlets were subjected to zero concentration constraints. Coupled CFD-transport systems were solved using COMSOL’s default solver. Maximum sample (fluorescein) height was calibrated using experimental data for a single set of sample and sheath flow rates, yielding a threshold concentration of fluorescein in the model. Fluorescein heights and widths were predicted by thresholding fluorescein concentration at the outlet. Particles used in tracing simulations were assumed to have a diameter of 6 um and density 1.002 kg/L, within the range of the parameters of a *Saccharomyces cerevisiae* cell [55]. Particles were subjected to Stokes’ drag, neglecting other force contributions. The simulation was initialized with 500 particles uniformly distributed at the sample inlet and traced for 10 ms. Particle positions were registered at the outlet and used to compute the mean particle position and its standard deviation for comparison with experiment.

### 2.4. Microscopy

To visualize flow focusing and particle confinement in these devices we used confocal microscopy (Leica TCS SP5 confocal microscope). Threshold was determined for the lowest-flowrate image using a modified version of the iterative intermeans algorithm as implemented in ImageJ (default method) [56]. This threshold was kept fixed for thresholding subsequent images. The concentration of microspheres (Fluoresbrite polychromatic red 6.0 micron, Polysciences Inc., Warrington, PA, USA) dispersed in fluorescein solution was 107 particles/ml. For bright-field imaging we used a Nikon Eclipse Ti-U inverted microscope. Images were acquired using a chromatic aberration free infinity (CFI) Plan Apo Lambda 60× oil objective (NA 1.4). The calculated lateral resolving power of the objective at 380 nm is 139 nm and the focal depth is 515 nm. A Point Gray Grasshopper (GS3-U3-23S6M-C) camera was used for image acquisition. Images of microspheres in flow were captured at 1330 frames/s and 7-μs exposure times. Images of yeast in flow were captured at 1000 frames/s and 5-μs exposure times.

Stationary images for neural network training were captured at the same conditions. Z-stacks were acquired at 0.25 µm offset between slices. Stacks were segmented automatically using a version of the Multiscale Adaptive Nuclei Analysis (MANA) algorithm adapted to bright-field image stacks [57]. Cell-containing frames were automatically detected by keeping images with maximum patch-wise variance at least twice as high as mean image variance. 128 × 128-pixel crops were extracted from cell-containing frames by locating the image-patch of maximum variance and cropping a square of 128 × 128 pixels around the patch center.

### 2.5. Microsphere Z-Displacement Regression

To determine the offset of each imaged bead from the focal plane, we used neural network-based regression. Z-stacks of static microspheres served as a training set (Supplementary File S1). A neural network was implemented in PyTorch (Figure S2a) [58]. It consisted of three convolutional blocks with leaky rectified linear unit activation [59,60], with batch normalization in every layer but the last. The network was trained on the z-stack data until convergence using the Adam optimizer with mean square error loss and initial learning rate 1 × 10^−4^ to predict the displacement of bead centers with respect to the focal plane [58,61]. Training images were augmented using random rotations, cropping and addition of Gaussian noise with mean 0 and standard deviation 0.1. Bright-field images of microspheres in flow were evaluated using the network and a z-displacement distribution of microspheres was computed.

### 2.6. Yeast Cell Z-Distance Regression

To determine the offset between yeast cell images acquired in flow and the focal plane, the strategy used for microspheres (see above) is not easily applicable, since yeast cells exhibit high variability in size and shape. Instead, we used pairs of bright-field cell images captured within the same field of view and at known z-distances from the focal plane. Z-stacks of stationary cells for all yeast species under consideration were acquired with an inter-slice spacing of 0.25 µm. Single-cell stacks were cropped from the acquired fields of view. Image augmentation (rotation, translation, mirroring, addition of noise) was used to yield visually different images at a known z-distance. Using this information, we trained a siamese neural network (Figure S2b) to predict the z-distance between pairs of single-cell images. A siamese neural network yields predictions for inputs relative to a reference by first embedding the reference and inputs using the same neural network module, concatenating the results of the reference-path and target-path, and finally applying a further neural network module for input-reference comparison [62]. This is done to discourage the neural network from learning to compare the input images pixel-by-pixel, instead of on a global scale. The siamese neural network was trained to infer the distance between pairs of z-stack slices for a single cell. Slices were augmented by random rotations, translations, zoom and Gaussian noise with mean 0 and standard deviation 0.1. The network was trained using the Adam optimizer with initial learning rate 1 × 10^−4^ until convergence [61]. Bright-field images of yeast cells in flow were embedded using the neural network. A well-focused *S. cerevisiae* cell was chosen as a reference for z-distance computation. Z-distances-to-reference were computed for all single-cell images to derive a z-displacement distribution.

### 2.7. Unsupervised Learning For Cellular Mixtures

To characterize a set of captured single-cell images, we use a probabilistic generative approach. We assume that for single-cell yeast images, *x* is drawn from a distribution *p_θ_*(*x*) = *p_θ_*(*x*|*z*) *p*(*z*) with parameters *θ* where *z* are low-dimensional latent variables, *p*(*z*) is the prior distribution of latent variables, and *p_θ_*(*x*|*z*) is the likelihood of an image *x* given a latent vector *z*. Here, *p_θ_*(*x*|*z*) is given by a neural network. Following [36], we construct a neural network to give a variational approximation *q_θ_*(*z*|*x*) to the true posterior *p*(*z*|*x*) by reparameterization and optimize the variational lower bound to the marginal log-likelihood log *p*(*x*) with respect to all neural network parameters *θ* (Figure S3). The neural network *q_θ_* maps single-cell images to samples from the low-dimensional latent distribution, and can thus be understood as an encoder, embedding data points into latent space. Similarly, *p_θ_* can be understood as a decoder, mapping samples from the latent distribution to high-dimensional single-cell images. Optimizing the variational lower bound is then realized as training the encoder and decoder to reconstruct input images well, under the constraint that the latent distribution should be as close as possible to the prior distribution *p*(*z*) (Figure S3, purple term). To successfully learn a latent space, where latent dimensions correspond to meaningful visual features (e.g., cell shape, cell focal plane), we follow and implement the FactorVAE term in the variational lower bound promoting the independence of latent dimensions (Figure S3, red term) [40]. This term penalizes the latent distribution’s total correlation (TC) as given by the Kullback–Leibler (KL) divergence of the marginal distribution *q*(*z*) and its corresponding factored distribution, which is a product of the distributions for each latent dimension [63]. This forces the latent distribution to be close to a product of independent distributions. Therefore, the neural networks are encouraged to learn a more strongly disentangled latent representation.

For our data set, a variational autoencoder with FactorVAE loss was trained on cell-containing crops (size 128 × 128 pixels) from continuous-flow imaging [41]. The encoder consisted of six convolutional kernels of size 3 × 3 with ReLU activation [59], and batch normalization with an increasing number of features (16, 32, 64, 128, 256, 512) [64], followed by reparametrization to yield a sample from a 10-dimensional normal distribution [36,65]. The decoder consisted of transpose convolutions in the reverse order of the encoder, again followed by ReLU activation and batch normalization. The discriminator used for FactorVAE loss computation was a multi-layer perceptron (MLP) with two layers, 64 hidden units and ReLU activation. The networks were trained using the Adam optimizer with initial learning rate 5 × 10^−4^, and factor loss balancing parameter γ = 10 until convergence. K-means clustering as implemented in scikit-learn was applied to the latent space to separate *S. cerevisiae* cells from *S. pombe* cells and compared to ground-truth species labels [66]. Nearest neighbors for sample cells were extracted using euclidean distance in latent space. The latent space was visualized in 2D using t-distributed stochastic neighbor embedding (TSNE) [67].

## 3. Results and Discussion

### 3.1. Simulation Results

**Figure 2:**
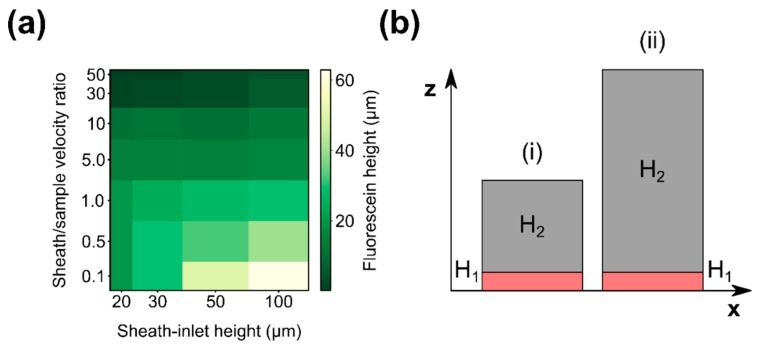
(**a**) Parametric sweep performed in COMSOL Multiphysics^®^. Flow velocity ratio (y-axis) refers to the ratio between sheath fluid velocity and sample flow velocity. Device height (x-axis) refers to the total height of the device, assuming a constant sample inlet height of 10 μm. Sample height (color scale) refers to the maximum distance between coverslip and sample. Log–log scale has been used to better resolve the areas of interest; (**b**) Cross sections of two-layer device geometries tested, where H_1_ is the height of the bottom layer and H_2_ is the height of the top layer: (i) H_1_ = 10 μm, H_2_ = 50 μm, (ii) H_1_ = 10 μm, H_2_ = 110 μm device.

Our goal is to use these microfluidic devices to deliver and confine yeast cells for in-flow imaging. Since cell diameter for yeast is typically 4–8 μm, we aim to confine sample flow within 10 μm from the microscope cover slide. To optimize our design, we used COMSOL Multiphysics^®^ to simulate the effect of device height and sheath-to-sample flow velocity ratio on the maximum distance between sample and microscope cover slide, referred to here as “sample height”. The results of the parametric sweep are shown in Figure 2a. According to the simulation, any sheath-to-sample flow velocity ratio over 20 (Figure 2a, y-axis) should result in sample height below 10 μm (Figure 2a, color scale, darker green color) relative to the coverslip. This appears to be independent of the height of the device used (Figure 2a, x-axis). To test the simulation results, we fabricated two devices with different heights, the cross sections of which are shown in Figure 2b. The first device has a total height of 60 μm (10 μm bottom layer height + 50 μm top layer height), and the second device has a height of 120 μm (10 + 110 μm).

### 3.2. Sample Confinement Testing Using Fluorescein

**Figure 3:**
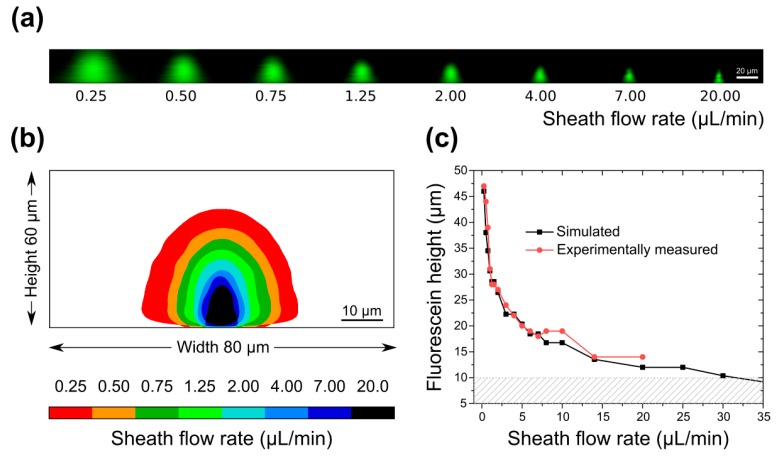
(**a**) Confocal microscopy images at increasing sheath-to-sample flow velocity ratio for 10 + 50 μm device. As sheath flow rate increases, fluorescein is confined both towards the microscope slide (bottom), as well as from the sides. In the z-direction the pixel size is w = 0.41 μm, h = 0.65 μm; (**b**) Z-projection of thresholded confocal microscopy images showing fluorescein confinement for increasing sheath flow rate; (**c**) Simulated (black) and experimentally measured (red) fluorescein heights for constant fluorescein flow rate of 0.25 μL/min and varying sheath flow rates. Shaded area highlights fluorescein height below 10 μm.

To visualize flow focusing in these devices we used confocal microscopy. Fluorescein dissolved in water was introduced through the sample inlet and was kept at a constant flow rate of 0.25 μL/min (0.87 mm/s). Water was used as sheath fluid and was introduced through the two taller, outer inlets at increasing flow rates. Figure 3a shows a montage of confocal images (channel cross section) for the device with a height of 60 μm. As predicted by simulation, for increasing sheath flow rates the volume occupied by fluorescein in the channel is progressively reduced. We observe fluorescein confinement in all directions, seen in the pictures as a reduction in the width and height of the fluorescein cone, also shown as an image overlay in Figure 3b. The reduction in the height of the fluorescein cone corresponds to confinement towards the microscope cover slip. When sheath flow rate is equal to sample flow rate (0.25 μL/min), fluorescein occupies a large fraction of the channel volume (red). With increasing sheath flow rates, the volume occupied by fluorescein shrinks towards the cover slip (black). The highest sheath flow rate achieved without affecting flow equilibrium was 20 μL/min, corresponding to a sheath-to-sample flow velocity ratio of 16. This is quantified in Figure 3c in a plot showing the measured fluorescein heights for every sheath flow rate tested. As seen in the graph, experimentally measured fluorescein heights correspond well with the equivalent values obtained from simulations, for sheath flow rates up to 20 μL/min. Since our final goal is to use optimized devices for applications in single-cell imaging in flow, sample flow rates were kept low in order to avoid image blur during imaging. For applications in flow cytometry, sample focusing can be optimized at higher flow rates.

Using these devices, we were not able to experimentally reproduce simulated sheath flow rates over 20 μL/min and fluorescein confinement below 10 μm was not achieved (maximum confinement was ~14 μm). Instead, for sheath flow rates over 20 μL/min (34.72 mm/s) we saw fluorescein backflow towards the sample inlet, suggesting a large difference in pressure between the sheath and sample inlets not predicted by the simulation shown in Figure 2a. To predict pressure driven backflow in microchannels using COMSOL Multiphysics^®^, pressure constraints need to be applied. For all simulations shown in this work, flow velocity constraints that prohibit backflow were applied instead. Indeed, when pressure constraints are applied instead of velocity constrains, we see a negative velocity in the x-direction within the sample channel, which confirms flow towards the sample inlet for sheath flow rates above 20 μL/min (Figure S4). These results demonstrate that devices with a height of 60 μm do not fulfill the necessary requirements for sample flow focusing within 10 µm from the coverslip.

**Figure 4:**
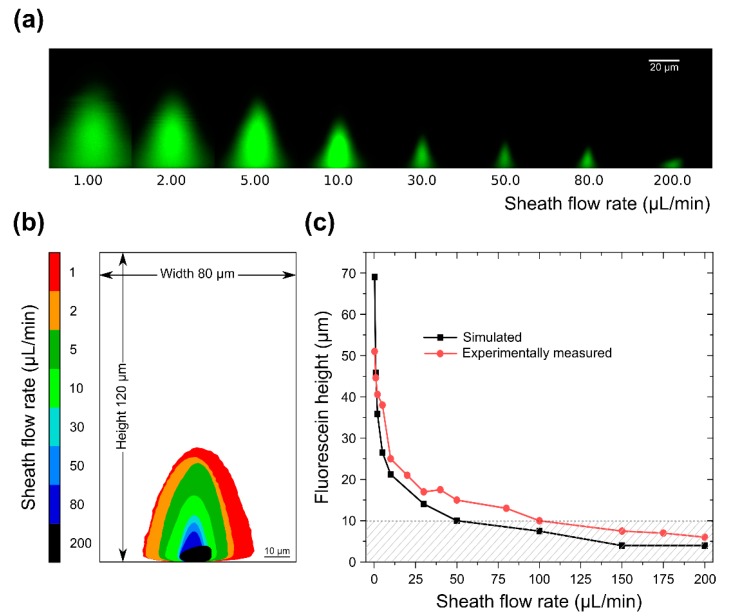
(**a**) Confocal microscopy images at increasing sheath-to-sample flow velocity ratio for the 120 µm (10 + 110 μm) device. In the z-direction, the pixel size is w = 0.43 μm, h = 0.43 μm; (**b**) Z-projection of thresholded confocal microscopy images showing confinement of the fluorescein cone for increasing sheath flow rate; (**c**) Simulated and experimentally measured fluorescein heights for constant fluorescein flow rate of 0.25 μL/min and varying sheath flow rates. Shaded area highlights fluorescein height below 10 μm, which is our target confinement height for live yeast cell in-flow imaging.

To eliminate the problem of sample backflow towards the inlet we next tested devices with a height of 120 μm. An increase in the area of the channel cross-section is expected to alleviate backflow since channel pressure is expected to drop. The logical way to achieve this would be to increase channel height, as opposed to channel width, as we would otherwise lose horizontal focusing. Similar to the previous device, the fluorescein flow rate was kept at 0.25 μL/min and sheath flow rate was slowly increased from 0.25 μL/min (0.43 mm/s) to 200 μL/min (347.22 mm/s). Again, to evaluate fluorescein confinement we used confocal microscopy and the results are shown in Figure 4a. We found that for a 1:1 sheath-to-sample flow rate ratio, fluorescein occupies a large fraction of the channel volume, also shown in red in the image overlay in Figure 4b. With increasing sheath flow rates, we observe continuous fluorescein confinement, seen in the figures as a reduction in the width and height of the fluorescein cone. Quantification of the fluorescein cone height from the images revealed a confinement within a distance of 10 μm from the cover slip at flow rates over 100 μL/min (173.61 mm/s), shown as the shaded area in the graph in Figure 4c. The distance between the microscope slide and the tip of the fluorescein cone decreased further to a minimum of ~5μm when the highest tested flow rate (200 μL/min) was used. At such high sheath flow rates, sample flow begins to become unstable, which results in a change in the observed fluorescein shape, as seen in Figure 4a. Importantly, due to the larger cross-section of this device, flow equilibrium was maintained, and no fluorescein backflow was present even at very high sheath-to-sample flow rate ratios. As seen in the graph, experimentally measured fluorescein heights correspond well with the equivalent simulated heights, and confinement below ~5 μm was experimentally reproduced. To better visualize confinement, we used COMSOL Multiphysics^®^ to generate animations that show sample flow confinement with increasing sheath flow rates in 2D and 3D, see Supplementary Animation S1 and S2. In summary, devices with a height of 120 µm can robustly confine fluorescein as close as ~5 μm from the microscope slide.

Since these devices are optimized for use in single-cell imaging in flow, it is important that flow focusing is maintained for several hundred micrometers after the junction in order to provide enough space for cell detection, cell imaging, and potential integration of cell sorting mechanisms [68]. We therefore investigated four positions within the device with increasing distance from the inlet junction; 100 μm, 500 μm, 1.1 mm, and 2.1 mm. Confocal microscopy images of fluorescein dissolved in water were taken at these positions and are shown in Figure S5i–iv. Flow equilibrium and sample confinement is maintained along the main channel even 2 mm away from the junction ensuring enough space for cell imaging and sorting. Even though the height of the fluorescein cone remains the same along the channel, fluorescein diffuses into the sheath fluid (water), which is to be expected in channels characterized by laminar flows where mass transport by transverse diffusion is the dominant mechanism for mixing [69,70].

### 3.3. Simulation of Particle Positioning and Validation Using Microspheres

**Figure 5:**
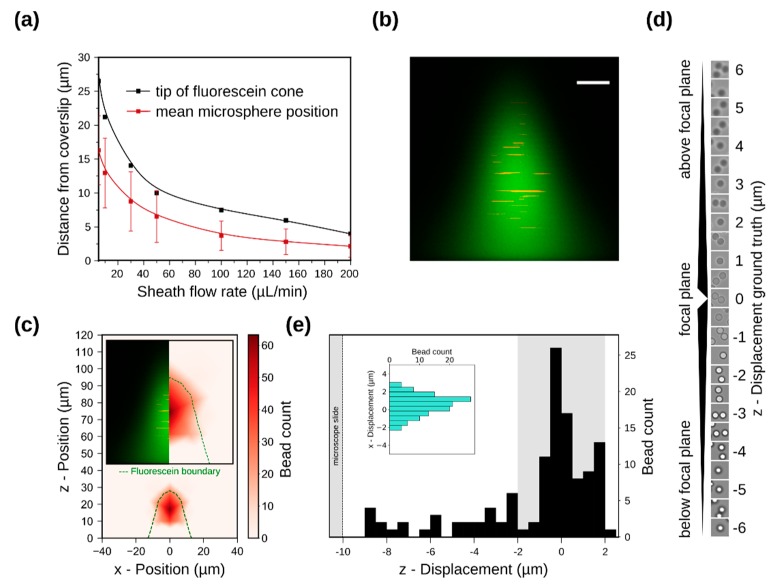
(**a**) Simulated distance between fluorescein cone tip and microscope slide for increasing sheath flow rates and fluorescein flow rate of 0.25 μL/min (black). Mean bead equilibrium position (along with standard deviation) with respect to the microscope slide (red); (**b**) Confocal microscopy image of the cross section of the sample stream (green), sheath fluid (black). Red fluorescent microspheres (diameter ~ 6 μm) dispersed in the sample stream appear as lines due to line scanning in confocal microscopy. This image was taken in a device with a height of 120 μm, at a fluorescein flow rate of 0.25 μL/min (0.43 mm/s) and sheath flow rate of 10 μL/min (17.36 mm/s). The height of the fluorescein cone in this image is 27 μm, and the scale bar is 6 μm; (**c**) Simulation reproducing microscopy data shown in (**b**). Fluorescein contour shown as green dotted line. Microspheres appear to concentrate closer to the fluorescein cone tip rather that the microscope slide. The insert shows a juxtaposition of the confocal image and the equivalent simulated data. (**d**) Microsphere images acquired at known distances from the focal plane, used as part of the training set for bead focal plane regression. (**e**) Histogram of z-displacement of microspheres relative to the focal plane. Bins have a width of 0.5 µm, with the x-axis the displacement relative to the focal plane and the y-axis the bead count for each bin. The shaded region within 2 µm of the focal plane, accounts for 68% of all microspheres. Displacement on the x-y plane is shown in the inset. 87% of sphere centers are located within 2 µm of the stream centerline.

For high-resolution imaging of cells flowing in these devices, it is important to be able to predict how cell position varies along the z-axis according to the flow rates used. To evaluate the equilibrium position of particles flowing in such channels, we first used simulations where we traced 500 microspheres with a diameter of 6 μm uniformly distributed at the sample inlet. We found that at low sheath flow rates, the mean equilibrium position of the microspheres (red squares in Figure 5a) is predicted to be closer to the tip of the fluorescein cone (Figure 5a, black squares) rather than the coverslip. In other words, the center of gravity of the microspheres is predicted to be on average closer to tip of the fluorescein cone than the coverslip. With increasing sheath flow rates (above 100 μL/min) and as the size of the fluorescein cone is decreasing due to flow confinement, the simulations predict that the mean equilibrium position of the microspheres will shift towards the microscope slide, close to the center of the fluorescein cone. This shift in the mean position of microspheres follows a shift in the flow streamlines, which is in turn based on an increase in the shear force exerted by the velocity gradient at higher flow rates (Figures S6 and S7). To validate the results from the simulations, we dispersed red fluorescent microspheres in fluorescein solution and used confocal microscopy to track their location. Due to the limited speed and the line-scanning mode of data acquisition in confocal microscopy, the moving microspheres appear as lines (Figure 5b,c). Even though the confocal microscope image in Figure 5b qualitatively confirms that particles equilibrate on average closer to the tip of the fluorescein cone rather than the base, a quantitative measurement of the position of microspheres is not possible due to limitations of the microscope. Simulations shown in Figure 5c, however, mathematically reproduce the microscopy data shown in Figure 5b. Again, most microspheres are predicted to reach equilibrium closer to the tip of the fluorescein cone (green contour) rather than the cover slip (bottom of the figure). To facilitate comparison between experimental and simulation data we included a juxtaposition of the two as an inset in Figure 5c.

To further quantify the distribution of microsphere positions within the sample phase without line-scanning artefacts, we used bright-field microscopy to image microspheres in flow at a fixed z-position in devices with a height of 120 µm. We set the imaging z-position to match the mean equilibrium z-position of microspheres predicted by simulation. Microspheres were dissolved in water at a concentration of 10^7^ particles/ml and were introduced into the device through the sample inlet. To keep the particle velocity within the range we can image without being affected by image blur, we used sample flow rate of 0.1 μL/min and sheath (water) flow rate of 10 μL/min. These flow rates resulted in microspheres being confined within 16 μm from the coverslip, with a mean equilibrium position at approximately 10 μm away from the coverslip. To automatically and accurately quantify the z-positions of all microspheres imaged in flow with respect to the microscope focal plane and the coverslip, we trained a simple neural network on z-stacks of images acquired from static microspheres (Figure 5d and Supplementary File S2). Details about the network can be found in Section 2.4. The distribution of microspheres within the sample stream is depicted as a histogram in Figure 5e. Microsphere z-displacement is shown relative to the true focal plane (z-displacement = 0), as well as relative to the microscope slide (z-displacement = 10 μm). Microspheres located within 2 μm from the focal plane, depicted by the shaded area in Figure 5e, account for 68% of all spheres imaged. As we already demonstrated in Figure 3 and Figure 4, further confinement is possible in these devices by increasing the sheath flow rate. It is expected that this will increase the percentage of microspheres located within 2 μm from the focal plane since there will be less space available for them to move. However, increasing the sheath flow rate also increases the velocity of the microspheres, which in turn results in significant motion blur that no longer allows us to quantify the microsphere distribution.

For applications in imaging flow cytometry, it is also important to ensure tight particle focusing on the x-y plane in order to minimize the field-of-view that is imaged and subsequently analyzed. The total width of the sample phase for the flow conditions described above was found to be ~10 μm. The displacement of bead centers from the stream centerline is provided as the inset in Figure 5e (green histogram). Almost all bead centers (87%) were found to lie within 2 μm from the sample stream centerline, which confirms excellent focusing along the x-axis and allows for the capturing of small images for fast read-out and processing. Together, our results demonstrate confinement of microspheres in a narrow stream close to the surface of the coverslip, as predicted by the simulations.

### 3.4. Single-Cell Imaging in Flow

**Figure 6:**
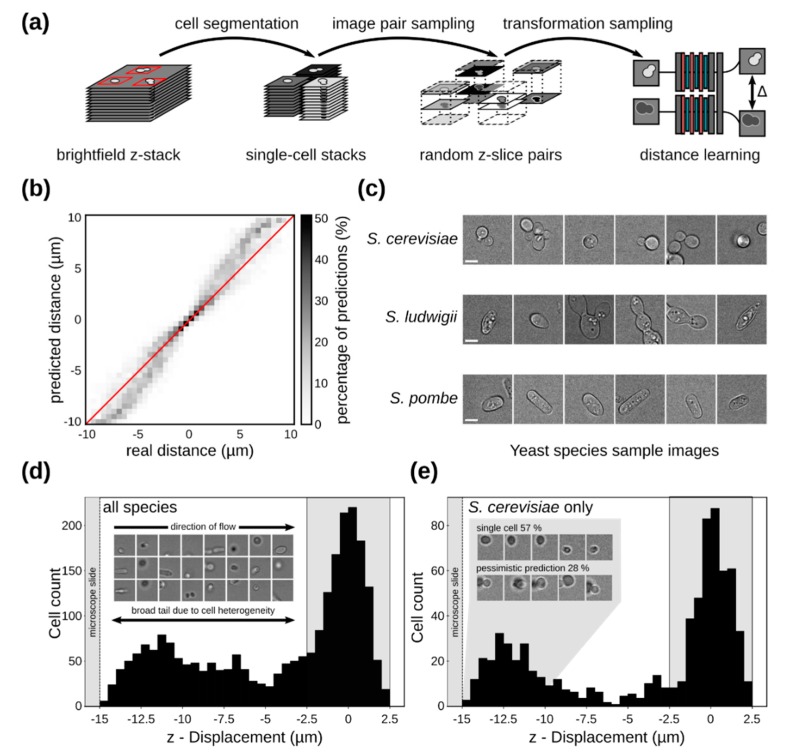
(**a**) Work flow underlying distance learning for automated z-position determination of flowing cells. Stacks of images of large fields of view containing many cells were recorded on the same microscope used for the imaging of flowing cells, but using an ordinary mounting of the cells. Single-cell stacks were cropped out. Random image pairs with known z-distances taken from single-cell stacks were used after image augmentation to train a Siamese neural network. (**b**) Predicted and real z-distance in pairs of cells taken from a test set of single cell stacks. (**c**) Images of the different yeast species used. (**c**) Example images of *S. cerevisiae*, *S. ludwigii* and *S. pombe* cells. The example cells highlight the high within-species and inter-species heterogeneity of the cells used. Scale bar length corresponds to 5 µm. (**d**,**e**) Histogram of z-displacement of flowing yeast cells in the device relative to an in-focus reference cell. Bins have a width of 0.5 µm, with the x-axis the z-displacement relative to the distribution median. Across all yeast species, example images highlighting their heterogeneity are shown. The shaded region within 2.5 µm of the focal plane contains 51% and 60% of imaged cells for all species (**d**) and *S. cerevisiae* (**e**) respectively. *S. cerevisiae* examples of non-budding cells and pessimistic predictions in the region below −10 µm are shown, accounting for 85% of the distributions lower peak.

Next, we replaced microspheres with yeast cells of different species, i.e., *S. cerevisiae*, *Sd. ludwigii* and *S. pombe.* Unlike microspheres, each of these species exhibit characteristic cell shapes in the size-range of 3–12 µm. *S. pombe* cells are rod-shaped, *Sd. ludwigii* are lemon shaped, and *S. cerevisiae* cells are round. Furthermore, cell shapes change through the life cycle since *S. pombe* divides by fission, while *Sd. ludwigii* and *S. cerevisiae* cells divide by forming a bud attached to the so-called mother cell. The irregularity in cell shape and sizes imposes limitations on the tools we can use to determine the z-displacement distribution of yeast cells within the focused sample stream in our device. The simple neural network training strategy used for microspheres (Section 2.4, Figure 5d) could not be used in this case due to the lack of homogeneity. Instead, we used pairs of bright-field cell images captured within the same field of view and at known z-distances from the focal plane. Single-cell stacks were cropped from the acquired fields of view and images were augmented to yield visually different images of cells with a known z-distance. Using this information, a siamese neural network was trained to predict the z-distance between pairs of single-cell images (Figure 6a) [62]. More details about yeast cell z-distance regression have been outlined in Section 2.5. To evaluate the learning success of this strategy, we used the trained network to predict the distance between pairs of images taken from a test data set that has not been used for training purposes. As shown in Figure 6b the neuronal network predicted with high accuracy the z-distance in the test data.

To continue with the analysis of cell focusing, we imaged yeast cells in flow under the same conditions described in Section 3.3 for microsphere imaging. Imaging data for all yeast species are given in Supplementary File S3. Some sample yeast images that highlight cell heterogeneity are shown in Figure 6c. We used bright-field microscopy to image cells in flow at a fixed z-position in devices with a height of 120 µm. The focal plane for yeast cells was found to be approximately 16 um from the coverslip. Using the trained neural network, we determined the position of each imaged cells within the device using well-focused cell images as reference. Similar to what we observed for microspheres in Figure 5e, the z-distribution for all species peaked around the focal plane of the microscope, with a broad tail towards the microscope slide (Figure 6d). When considering cells of all species and sizes, the main peak around the focal plane (z-displacement = 0) contained 51% of imaged cells. The broad tail formed at the bottom of the device, adjacent to the microscope slide can be explained by the broad heterogeneity of the sample, since size and shape distributions are known to influence particle focusing positions in different types of devices [71,72]. Given this information, and in addition to the fact that the tail is much less pronounced when imaging monodisperse microspheres under identical flow conditions in the same devices, we assume that this broad tail is in fact due to cell heterogeneity, examples of which are shown in the inset of Figure 6d. To test this assumption, we limited cell-shape degrees of freedom by considering only *S. cerevisiae* cells in Figure 6e. The distribution now clearly shows two peaks, where the main peak contains 59% of all cells. Closer examination in the composition of the lower peak reveals that 57% of cells are small, single cells and 28% are budding cells. These cases are susceptible to pessimistic neural network predictions. Here, we call a network prediction pessimistic, when the maximum possible z-distance between the imaged cells and the reference cell is predicted. For example, if an input image contains a well-focused mother cell and an out-of-focus daughter cell, the network uses the z-distance between the out-of-focus daughter cell and reference cell, and therefore classifies the entire image as out-of-focus, even though the mother cell is in focus. Examples of these events are shown in the inset of Figure 6e. In total, 85% of lower-peak cells are either differently sized than the main-peak cells, or have pessimistic z-distance predictions. For the purposes of evaluating the cell focusing performance of our device compared to that of microspheres of the same size, we may therefore safely neglect the z-distribution’s lower peak. This indicates that the simple microfluidic devices we developed can be successfully used to also focus cells, allowing for imaging and downstream tasks, such as sample characterization and cell sorting.

### 3.5. In-Flow Cell Imaging and Cell Classification

Imaging large cell populations using conventional microscopy requires scanning using large mechanical moving stages. Imaging flow cytometry offers the possibility of imaging many cells while avoiding moving mechanical parts. Moreover, each cell is imaged individually, eliminating the need for retrospective cell segmentation and object identification in large fields of view, which is often a daunting task especially when cells are close together. Microfluidic devices like the one described in this work offer the possibility for high-throughput imaging and open up applications that also involve rapid, in-line characterization of the investigated sample. To outline possible applications, we took imaging of different yeast species within our devices another step further and concluded this work by devising a flexible framework for efficient, automated imaging data analysis. To this end, we implemented unsupervised machine learning in the form of a VAE to extract phenotypic information from single-cell images without the need for user intervention.

**Figure 7:**
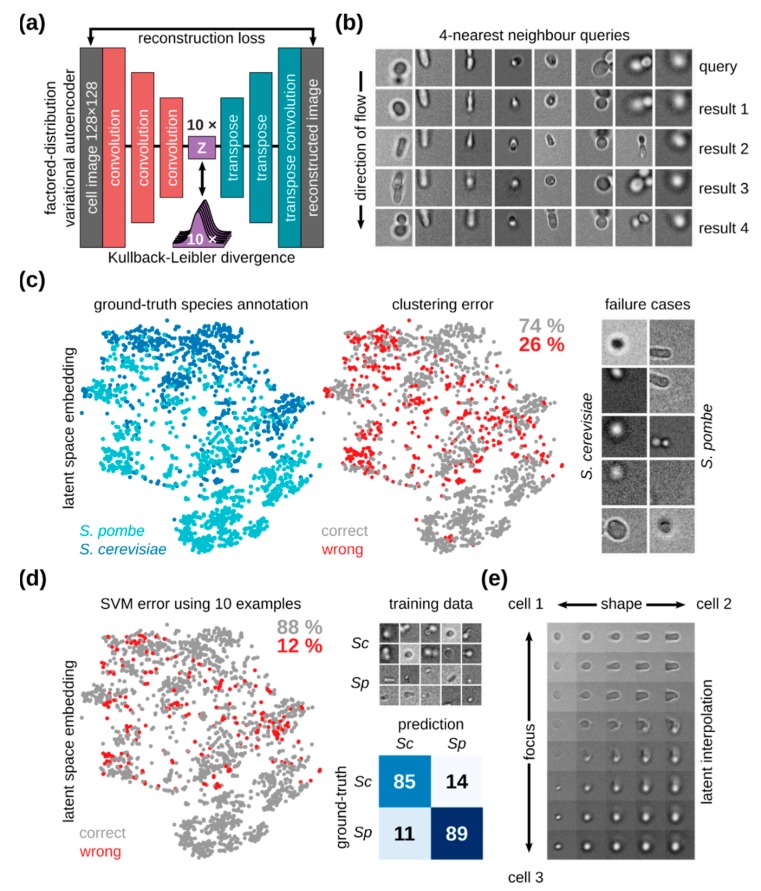
(**a**) Variational autoencoder (VAE) architecture for unsupervised learning. Cell images are convolutionally embedded into 10-dimensional latent space and reconstructed using same-shaped transpose convolutions. The network is trained to perform image reconstruction and is constrained to produce a disentangled latent space by KL divergence relative to a normal distribution and a penalty on total correlation. (**b**) Sample nearest neighbor queries for eight query cells. Query results are displayed in the order of increasing distance in latent-space. (**c**) Assessment of unsupervised classification accuracy. A two-dimensional embedding of data points for *S. cerevisiae* (blue) and *S. pombe* (turquoise) is shown, with ground truth species labels (left), a map of data points wrongly classified (red) by latent space k-means (center), and images of random failure cases for both species (right). Failure cases comprise *S. cerevisiae* cells classified as *S. pombe* cells (left column) and vice versa (right column). k-means on latent space classifies 74% of samples correctly, without the need for supervision. (**d**) Assessment of few-shot classification accuracy. A map of wrongly classified data points (red) using an support vector machines (SVM) classifier on latent space with 10 training examples per species shows an accuracy of 88% (left). The full training set is displayed for both species (top right), together with a confusion matrix showing the percentage of classifications for the classifier (bottom right). *Sc* and *Sp* indicate *S. cerevisiae* and *S. pombe* respectively. (**e**) Latent space interpretability. A latent space interpolation between three cells is shown, indicating latent space vectors encoding for cell focal plane (focus), as well as cell elongation (shape).

To demonstrate the capabilities of our framework, we trained a VAE with FactorVAE loss on single-cell images (see Section 2.6) and classified yeast cells by species in a fully unsupervised manner. The model consists of an encoder and a decoder. The encoder maps images to 10-dimensional points in latent space, while the decoder generates images from such points. A schematic of the model and objectives involved is shown in Figure 7a. The model is trained to have generated images match input images and produce a latent space, where each dimension corresponds to a meaningful visual characteristic of cells. For example, the cell species should vary along one dimension, while the cell shape should vary along another (for more details, please refer to Section 2.6). Qualitative evaluation of the resulting latent space by nearest neighbor analysis shows that single-cell images at a similar level of focusing and of similar shape are close together in the learned representation. As such, the four nearest neighbors shown in Figure 7b are budding *S. cerevisiae* cells, if the query cell is a budding *S. cerevisiae* cell, and elongated cells, if the query is an elongated cell. This suggests that our VAE has learned a meaningful latent representation, which could be applied to downstream classification tasks.

As our VAE learns visual similarities and differences among imaged cells, a high within-species variance of cells increases the difficulty of distinguishing different species. One factor resulting in high intraspecies variance is a lack of cell focusing. On the other hand, images of similarly sized, well-focused cells reduce intraspecies variance and as a result greatly simplify and improve classification. Our device achieves such improvements by precisely focusing similarly sized cells close to the microscope slide. In addition to particle focusing along the z-axis, our device tends to orient non-spherical particles in the direction of fluid flow, further reducing intraspecies variance. This tendency can be seen in the cells displayed in Figure 7b. Our model learns this regularity of the data and therefore groups similarly oriented cells.

To allow for the automated analysis of data from our devices, the learned representation should capture phenotypic properties necessary for distinguishing single-cell images of different yeast species. We evaluated the suitability of our learned representation for this task of unsupervised classification by using it to distinguish between images of *S. cerevisiae* and *S. pombe* cells captured in-flow in our microfluidic devices. *S. ludwigii* cells were not used for classification since they tend to form aggregates that disrupt flow and clog the microfluidic channels, resulting in few available examples of *S. ludwigii* cells in flow compared to *S. cerevisiae* and *S. pombe*. A qualitative inspection of the latent space labeled by yeast species (Figure 7c, left) reveals a separation between budding and fission yeast cells, with similar subsets of cells forming smaller clusters. Fully unsupervised classification is performed with an accuracy of 74%. Data-points that were wrongly classified (Figure 7c, center) are mostly located in regions where species labels overlap. Some failure cases are shown on the right in Figure 7c. A qualitative inspection of these failure cases reveals small out-of-focus *S. cerevisiae* cells being misclassified as *S. pombe* cells, while round out-of-focus *S. pombe* cells are misclassified as *S. cerevisiae* cells. Both of these are cases of misclassification, where distinguishing between yeast species becomes hard even for human experts. We have therefore achieved fully unsupervised classification of yeast cell by species with an accuracy of 74%. This complements our device with a strong baseline for cell classification without the need for expert intervention.

While fully unsupervised classification removes the need for large, hand-annotated training data sets, annotating small amounts of training examples is both feasible and beneficial for classification accuracy. We extend our data analysis setup with few-shot cell classification, to improve accuracy and enable expert-guided adaptation to more fine-grained classification tasks. To this end, we trained an SVM classifier on a representative set of 10 annotated single-cell images per species (Figure 7d, top right). The few-shot classifier increases classification performance to an accuracy of 88%, with negligible training time (Figure 7d). While misclassified data-points occur at similar locations in latent space as for fully unsupervised classification, the number of wrongly classified cells is significantly reduced (Figure 7d, left). The confusion matrix for the SVM classifier (Figure 7d, bottom right) shows that both *S. cerevisiae* and *S. pombe* cells are misclassified as the other with a probability of 11–14%, a vast improvement compared to unsupervised classification. Our integrated platform can therefore perform few-shot classification of yeast cell images captured in-flow in our microfluidic device and achieve 88% success in separating yeast cells by species.

In flow cytometry, it is desirable to gate single-cell events by their properties, e.g., fluorescence intensities. In imaging flow cytometry, such properties correspond to spatially resolved features, such as cell morphology and subcellular protein localization. Ideally, our latent space should capture those properties. Qualitative inspection of the latent space reveals interpretable latent dimensions, which can be linked to biologically meaningful morphological features, such as cell focal plane and elongation. These are visualized in a latent interpolation between cells along these dimensions in Figure 7e. The results in this figure also show that our latent space captures valuable semantic information, which could be used to differentiate between different cell phenotypes based on morphology in bright-field images. These indicate that our combined microfluidic and unsupervised learning platform can be used for the biologically-relevant characterization of complex cellular mixtures with minimal human intervention. The learned semantic latent space captures enough phenotypic features to group cells based on their morphology and extract accurate subpopulation classifiers with very few training examples.

## 4. Discussion

In this work, we have demonstrated 3D flow focusing in simple microfluidic devices for applications in imaging flow cytometry. The devices used utilize a difference in height between the outer sheath inlets and the middle sample inlet and achieve sample flow confinement within a few micrometers from the microscope slide, which makes them suitable for use with high NA oil objectives. In contrast to most previously demonstrated geometries, our devices maintain a simple, single-layer architecture that makes them accessible to non-expert users. Instructed by simulations, we fabricated and tested two devices with different heights, 60 μm and 120 μm, and found that flow equilibrium between sheath and sample is more stable in taller devices, and sample confinement within 5 μm from the microscope slide was achieved. To evaluate device performance for use in single-cell imaging in flow, we introduced 6-μm polymer microspheres dispersed in fluorescein through the sample inlet and monitored their position using confocal microscopy. Further, we used simulation tools to predict bead equilibrium positions in these devices for different sheath flow rate ratios and the results were confirmed using confocal microscopy. Once the mean microsphere equilibrium positions had been calculated and confirmed, we used bright field microscopy to image fast-moving beads traveling through these devices. We applied a novel, neural network-based approach to determine the distance of spheres to the focal plane. We found that 68% of the microspheres were traveling within 2 μm from the focal plane, thus enabling the acquisition of single plane images across their body. These results were reproduced for yeast cells of multiple species travelling in the same devices, further demonstrating the applicability of such devices in imaging flow cytometry. Our optimized microfluidic device can be used for a range of flow rates (0.1 μL/min–200 μL/min have been tested) and a range of particle sizes (3–12 um tested) without the need for geometry modification, which makes it robust and compatible with both imaging and non-imaging flow cytometry. Additionally, such devices could be relevant in other applications where hydrodynamic focusing into a small, well-defined volume is required [73].

Furthermore, we applied state-of-the-art unsupervised learning techniques to classify yeast cells by species. This was done in a fully unsupervised manner and also using SVM in a few-shot setting. We learned a semantically meaningful latent representation of yeast cells, with latent vectors representing visually and biologically meaningful features. Our latent representation allowed for the unsupervised distinction between *S. cerevisiae* and *S. pombe* cells with 74% accuracy, increasing to 88% in the 10-shot setting, which proves it suitable for further downstream classification tasks. We also verified via nearest-neighbor analysis, that biologically and visually similar cells are grouped in latent space. In summary, we have presented the first application of unsupervised learning in imaging flow cytometry. Interpretable latent spaces provide biologically meaningful image parameters that could improve image-activated cell sorting and allow for FACS-like gating on imaging data.

Our demonstration only utilized bright field imaging of microspheres and yeast cells. For practical applications, imaging flow cytometry requires the capability to acquire fluorescent images of cells. This requires acquisition speeds that are fast enough to reveal subcellular structures inside the moving cells, ideally in the range of the diffraction limit of a high NA objective (i.e., ~200 nm lateral and ~800 nm vertical resolution when using a 1.4 NA oil objective). This poses several challenges, some of which can be overcome using different techniques already described in the literature [6].

## 5. Conclusions

In conclusion, we have demonstrated a learning microfluidic platform capable of imaging live cells in flow and classifying acquired images without the need for human supervision. Cell streaming, confinement and imaging are achieved in a simple and versatile microfluidic device. Such devices can confine flow towards the microscope slide in a controlled manner, which makes them especially suitable for applications in single-cell imaging in flow. We have demonstrated that a large range of flow rates and particle sizes can be used without the need for geometry modification, since both tightness of focusing and line of focusing along the z-direction only depends on the flow rate ratio between sheath and sample. In fact, we were able to successfully stream, focus, and image *S. cerevisiae*, *S. ludwigii* and *S. pombe* yeast. This is the first demonstration of controlled 3D flow confinement well below the inlet height in this simplified version of a single layer Y-shaped microfluidic device and the firsttime particle and cell positioning in the focused stream are studied. We expect that such a robust device will find applications in the quickly growing fields of imaging flow cytometry and flow cytometry on-chip.

Additionally, we achieved image classification using a powerful unsupervised learning paradigm of disentangling variational autoencoders. Our variational autoencoder embeds single-cell images in an interpretable latent space and allows for both similarity-based queries and classification. The biggest advantage of such classification is that it is completely unsupervised, obviating the need for large hand-annotated training data sets prevalent in neural network-based machine learning. To our knowledge, this is the first application of unsupervised representation learning to imaging flow cytometry. In particular, disentangled representation learning has not been applied to single-cell images before and we expect it will play a big role in gating for image activated cell sorting. In conclusion, we presented a simple and affordable platform for continuous-flow single-cell imaging, large-scale data analysis, and image classification.

## Supplementary Materials

The following are available online at https://www.mdpi.com/2072-666X/10/5/311/s1, Figure S1: Device fabrication schematic, Figure S2: Schematic of neural network architectures, Figure S3: Schematic of variational autoencoder training, Figure S4: X-direction velocity profile simulation for the 60 µm (10 + 50 µm) device, Figure S5: Flow focusing at four positions within the device with respect to the junction, Figure S6: Qualitative shear force distribution due to xy-velocity gradients in the z direction, Figure S7: Theoretical flow focusing behavior dependent on x-velocity, Supplementary Animation S1, Supplementary Animation S2. Supplementary Files S1–S3 are deposited in the heiDATA Dataverse repository at: https://doi.org/10.11588/data/L5J7WO. Source code and neural network weights for all parts of the project are deposited on GitHub at: https://github.com/mjendrusch/learning-ifc.
