# Supplementary material for: Self-Learning Microfluidic Platform for Single-Cell Imaging and Classification in Flow
Source: Micromachines (Basel). 2019 May 9;10(5):311. doi: 10.3390/mi10050311 (PMC6563144; doi:10.3390/mi10050311)
Supplement: Supplementary file 1 [file micromachines-10-00311-s001.zip › micromachines-502301-SI/supplementary.pdf]

micromachines

*Article*

# Self-Learning Microfluidic Platform for Single-Cell Imaging and Classification in Flow

**Iordania Constantinou [1,2,3,†,*], Michael Jendrusch [1,†], Théo Aspert [4,5,6,7], Frederik Görlitz [1], André Schulze [1], Gilles Charvin [4,5,6,7] and Michael Knop [1,8,*]**

1 Zentrum für Molekulare Biologie der Universität Heidelberg (ZMBH), DKFZ-ZMBH Alliance, Universität Heidelberg, 69120 Heidelberg, Germany; jendrusch@stud.uni-heidelberg.de (M.J.);f.goerlitz@gmx.net (F.G.); a.schulze@zmbh.uni-heidelberg.de (A.S.)
2 Institute of Microtechnology, Technische Universität Braunschweig, 38124 Braunschweig, Germany
3 Center of Pharmaceutical Engineering (PVZ), Technische Universität Braunschweig, 38106 Braunschweig, Germany
4 Developmental Biology and Stem Cells Department, Institut de Génétique et de Biologie Moléculaire et Cellulaire, 67400 Illkirch-Graffenstaden, France; aspertt@igbmc.fr (T.A.), charvin@igbmc.fr (G.C.)
5 Centre National de la Recherche Scientifique, 67400 Illkirch-Graffenstaden, France
6 Institut National de la Santé et de la Recherche Médicale, 67400 Illkirch-Graffenstaden, France
7 Université de Strasbourg, 67400 Illkirch, France
8 Cell Morphogenesis and Signal Transduction, German Cancer Research Center (DKFZ), DKFZ-ZMBH Alliance, 69120 Heidelberg, Germany
* Correspondence: i.constantinou@tu-braunschweig.de (I.C.); m.knop@zmbh.uni-heidelberg.de (M.K.); Tel.: +49-0531-391-9769 (I.C.); +49-6221-54-4213 (M.K.)
† These authors contributed equally to this work.

**Contents**

**1. Supplementary Figures**

**2. Supplementary Methods and Theory**

*2.1. Device Physics*

*2.2. Neural Network*

## 1. Supplementary Figures

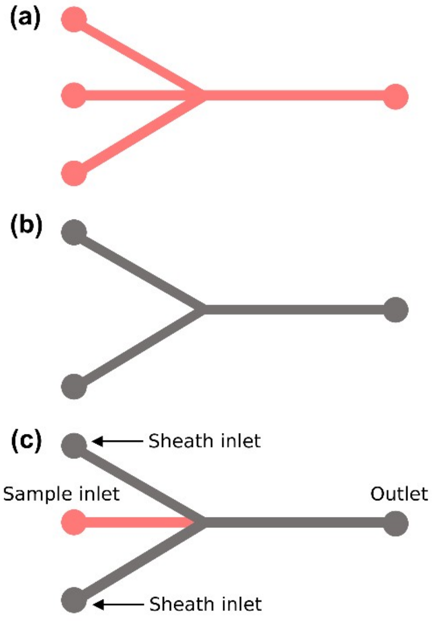


**Figure S1.** Device fabrication schematic. Top view of: (a) bottom layer of photoresist, (b) top layer of photoresist, and (c) full device.

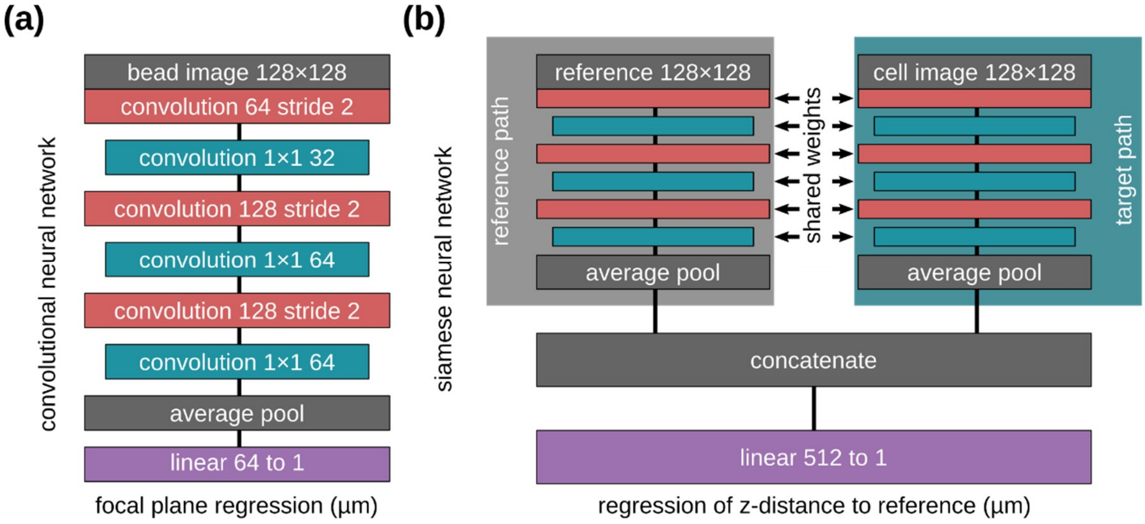


**Figure S2.** (**a**) Convolutional neural network architecture for regression of bead z-displacement to the focal plane. Convolutional blocks have kernel size 3×3, stride 1, leaky ReLU activation and batch normalization in every block but the last, unless otherwise specified; (**b**) Siamese neural network architecture for regression of yeast z-distance to reference. Reference images are embedded using a neural network block along the reference path, while target images are embedded along the target path. The reference and target embeddings are concatenated and a linear transformation produces the final regression result.

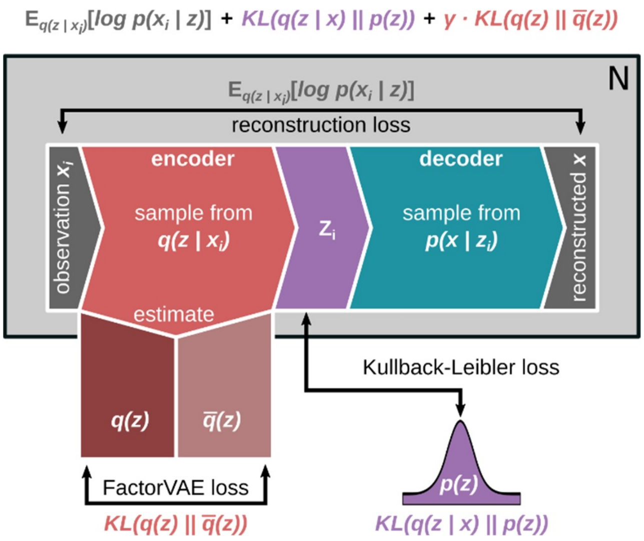


**Figure S3.** Variational autoencoder training. For a batch of training data (observations) with size N, data are encoded and samples zi are drawn from the variational posterior. The total correlation **(red)**, Kullback-Leibler divergence to the prior **(purple)** and the reconstruction loss **(grey)** are estimated from the density ratio of all samples in the batch. The encoder and decoder networks are trained by minimizing the weighted sum of these, as shown at the top. Here, ***KL(p || q)*** denotes the Kullback-Leibler divergence of two distributions.

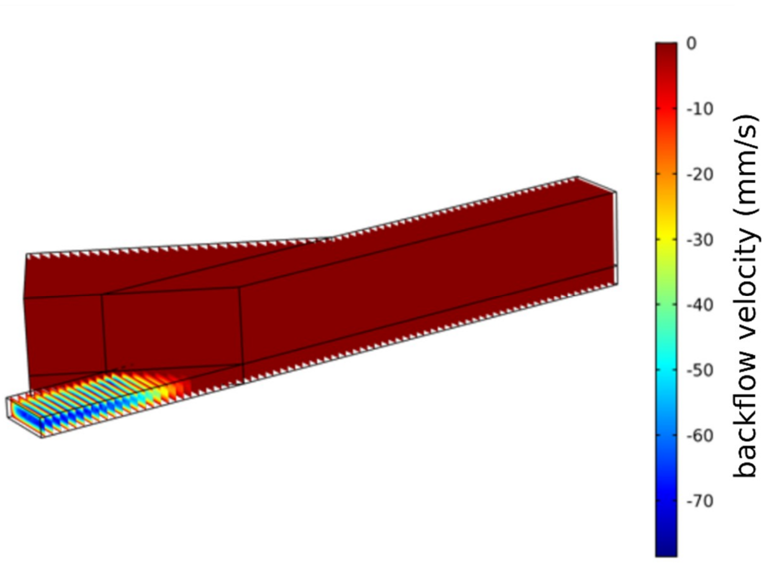


**Figure S4.** X-direction velocity profile simulation for the 60 μm (10 + 50 μm) device, constrained to velocities less than zero, with flow velocity constraints (sample flow 0.25 μL/min, sheath flow 50 μL/min) substituted by comparable pressure constraints. Negative x-direction flow, i.e. backflow with flow velocities on the order of 70 mm/s can be observed at the sample inlet. Backflow is not predicted in simulations using flow-velocity constraints, as those constraints fully specify the direction and velocity of flow through their inlets.

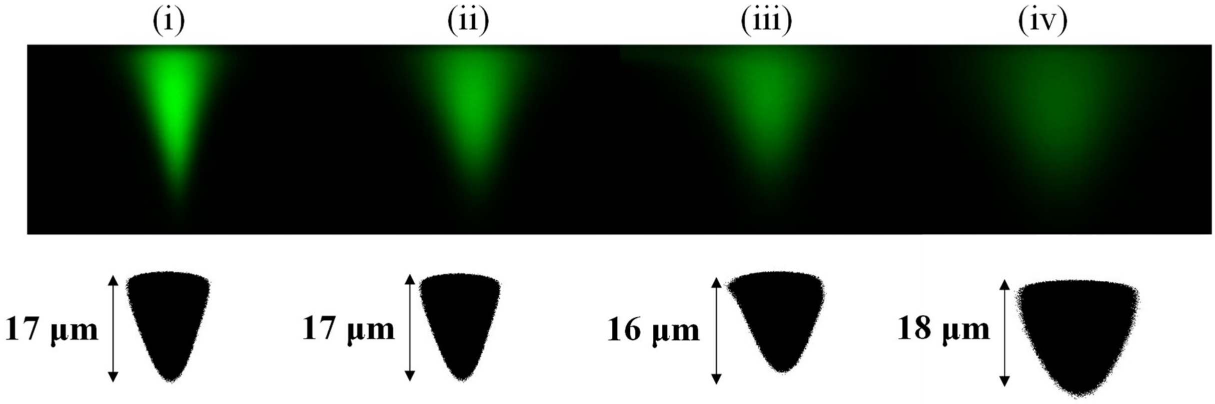


**Figure S5.** Flow focusing at four positions within the device with respect to the junction. (i) 100 µm away, (ii) 500 µm away, (iii) 1.1 mm away, (iv) 2.1 mm away. (Top) Confocal microscopy images at these positions. (Bottom) Equivalent thresholded images along with the measured fluorescein height.

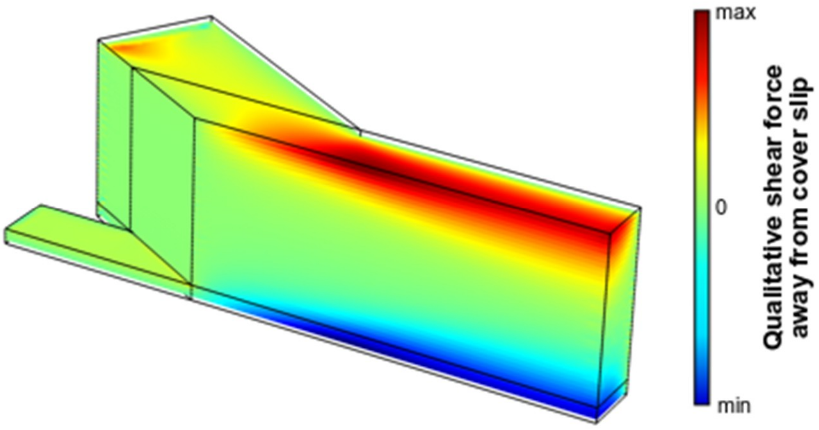


**Figure S6.** Qualitative shear force distribution due to xy-velocity gradients in the z direction, assuming zero z-velocity for a 120 m (10 + 110 µm) device at sample flow-rate 0.25 µL/min and sheath flow rate 100 µL/min, displaying the downward force causing flow focusing. Magnitude of downward force increases from dark red (minimum) through green (zero) to dark blue (maximum).

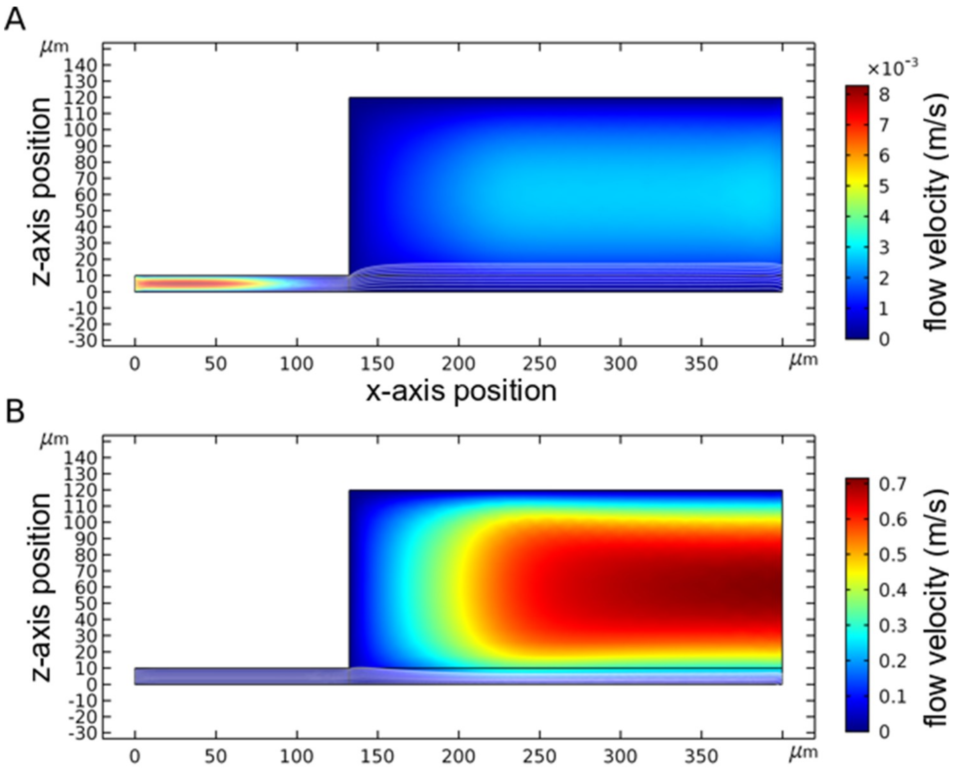


**Figure S7.** Theoretical flow focusing behavior dependent on x-velocity on the symmetry-plane of a 10 + 110 μm device at a sample flow rate of 0.25 μL/min and different sheath flow rates. (A) Streamlines for sheath flow rate 0.25 μL/min show defocusing away from the coverslip in accordance to the shear force exerted by the velocity gradient. (B) Streamlines for sheath flow rate 100 μL/min show focusing towards the coverslip in accordance to the shear force exerted by the velocity gradient.

## 2. Supplementary Methods and Theory

### 2.1. Device Physics

Given the device of Figure 1, consider an incompressible Newtonian fluid inside the device and laminar inflow at the inlets. Given a volume element $dV$ within the device moving with velocity projected unto the xy-plane $u_{xy}(x, y, z)$. It is subjected to a shear force in the z-direction:

$$F_z = \int \mu\, \partial_x \partial_z u_x(x, y, z) + \partial_y \partial_z u_y(x, y, z) dV$$

$$= \mu \left( \Delta A_{xz} \partial_z u_y(x, y, z) + \Delta A_{yz} \partial_z u_x(x, y, z) \right)$$

Thus, the volume element experiences a downward force if its velocity in the xy-plane is higher than that of the element below it, generating a z-direction force profile as shown in Figure S6. This in turn causes a downward flow resulting in y-dimension focusing towards the cover slip. For this to happen, sheath flow velocity at the height of the sample inlet has to be higher than sample flow velocity at that height, when exiting the sample inlet, requiring sheath flow rate to be much higher than sample flow rate, as seen in Figure S7. Otherwise, focusing towards the cover slip cannot be observed.

### 2.2. Neural Network for Bead z-Displacement Regression

A shallow neural network consisting of three convolutional blocks with batch normalization and leaky rectified linear unit activation, followed by global averaging and a final linear layer was set up as seen in Figure S2a. The neural network was trained on a dataset comprised of 822 random crops of the same 6 μm beads used in experiment, at defined z-positions relative to their focal plane,

ranging from -8 µm to 8 µm in steps of 0.5 µm. The images were normalized to mean zero and standard deviation one. To prevent rapid overfitting, the dataset was augmented by random cropping, rotation and gaussian noise with mean zero and standard deviation 0.1. The neural network was trained for 350 steps (1090 epochs, or until convergence) using the Adam optimizer at learning rate 0.001 with a constant learning rate schedule and batch size 128. Training was performed with the PyTorch library using synchronous stochastic gradient descent on 20 processes.

### *2.2.1. Siamese Neural Network for Yeast z-Distance Regression*

A densely connected neural network consisting of five convolutional blocks and a final convolutional output layer was implemented in PyTorch. Each convolutional block consisted of a 3×3 convolutional kernel, followed by a 1×1 convolutional kernel and another 3×3 kernel. ReLU activation and batch normalization were applied after each convolution. All convolutional blocks but the first used depth-wise separable convolutions for parameter reduction. The output of each convolutional block was concatenated with its input and fed into the next convolutional block. The network was applied to pairs of single, or dividing cell crops of yeast cells. Results were concatenated and fed into a final linear layer for distance regression (Figure S2b). The neural network was trained on a dataset comprised of multiple acquired fields of view of imaged *S. cerevisiae, S. ludwigii* and *S. pombe* cells, cropped to 128×128-pixel images containing single or budding cells. The neural networks were trained using the Adam optimizer at learning rate 0.001 with a constant learning rate schedule and batch size 128 until convergence. Training was performed with the PyTorch library using synchronous stochastic gradient descent on an NVidia GTX 1080 GPU.
