# Supplementary figures and images for: Self-Learning Microfluidic Platform for Single-Cell Imaging and Classification in Flow
Source: Micromachines (Basel). 2019 May 9;10(5):311. doi: 10.3390/mi10050311 (PMC6563144; doi:10.3390/mi10050311)

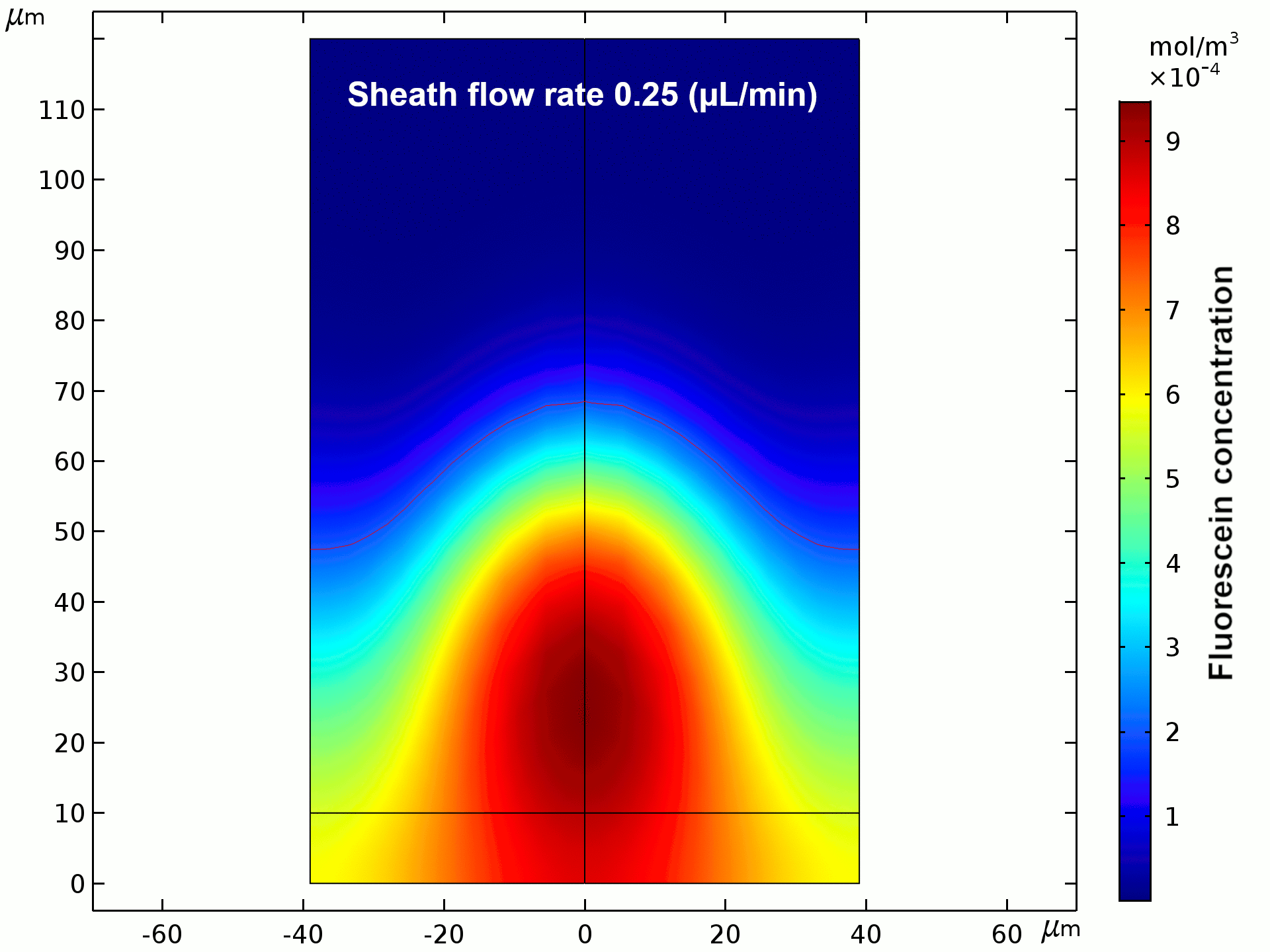

Supplement: Supplementary file 1 [file micromachines-10-00311-s001.zip › micromachines-502301-SI/Supplementary Animation S1.gif]

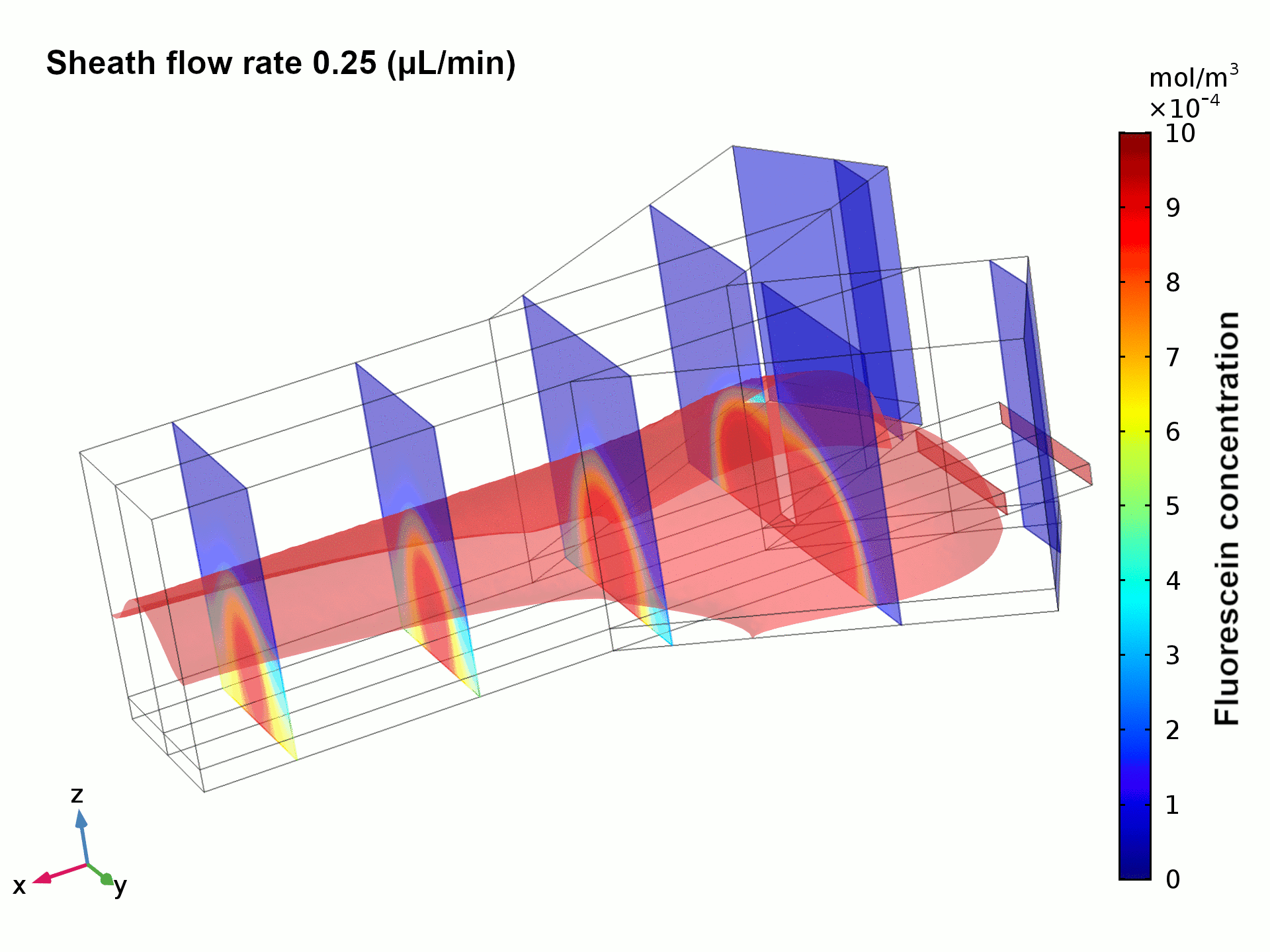

Supplement: Supplementary file 1 [file micromachines-10-00311-s001.zip › micromachines-502301-SI/Supplementary Animation S2.gif]
